# Supplementary material for: Effect of a Multi-Ingredient Coriolus-versicolor-Based Vaginal Gel in Women with HPV–Dependent Cervical Lesions: The Papilobs Real-Life Prospective Study
Source: Cancers (Basel). 2023 Jul 29;15(15):3863. doi: 10.3390/cancers15153863 (PMC10417075; doi:10.3390/cancers15153863)
Supplement: Supplementary file 1 [file cancers-15-03863-s001.zip › cancers-2356173-supplementary.pdf]

Table S1. List of the different centres that took part in the study and the location

Centre	Location
CLÍNICA ISIDRO LAGO	Pontevedra
HOSPITAL QUIRÓN A CORUÑA	A Coruña
CENTRO GINECOLÓGICO DE LEÓN	León
CLÍNICA EUSKALDUNA	Bilbao
CLÍNICA VIAMED-ALXEN	Logroño
INSTITUTO FONTSASTRE	Mataró (Barcelona)
HOSPITAL GENERAL DE CATALUNYA	St Cugat del Vallés (Barcelona)
BUFETMEDIC HOSPITALET	Hospitalet de Llobregat (Barcelona)
CLÍNICA VEGA MEDIA	Molina de Segura (Murcia)
CLÍNICA PRIVADA DR. OLALLA	Málaga
CLÍNICA GINEMED	Sevilla
CLÍNICA PRIVADA DR. RODRÍGUEZ MARTÍN	Algeciras (Cádiz)
INSITUTO PALACIOS DE SALUD DE LA MUJER	Madrid
HOSPITAL SANITAS LA ZARZUELA	Madrid
MD ANDERSON	Madrid
HM GABINETE VELÁZQUEZ	Madrid
HOSPITAL QUIRÓN MÁLAGA	Málaga
HOSPITAL MANACOR	Manacor (Baleares)
HOSPITAL UNIVERSITARI I POLITÈCNIC LA FE	Valencia
HOSPITAL UNIVERSITARIO VIRGEN DE LAS NIEVES (FIBAO)	Granada
HOSPITAL CLÍNICO SAN CARLOS	Madrid
HOSPITAL UNIVERSITARIO INFANTA LEONOR	Madrid
HOSPITAL GENERAL UNIVERSITARIO ALICANTE	Alicante
HOSPITAL SANTA ÁNGELA DE LA CRUZ	Sevilla
HOSPITAL JUAN RAMÓN JIMÉNEZ	Huelva
GINECOM	Las Palmas de GC
HOSPITAL NTRA SRA ROSARIO	Madrid
CLÍNICA ALBORÁN CMM	Almería
BONAL GINECOLOGÍA	Granada
Dr. Rosa María Fernández Arguelles. Private practice	Langreo (Asturias)
Dr. Lucía Vior Martínez. Private practice	Oviedo

CLÍNICA BUENAVISTA	Oviedo
CENTRO MÉDICO ASTURIAS	Oviedo
HOSPITAL UNIVERSITARIO VIRGEN DE VALME	Sevilla
CENTRO MÉDICO PALENCIA	Palencia
AVANTMÈDIC	Lleida
Dr. Perla Hernández Muñoz. Private practice	Mieres (Asturias)
FUNDACIÓN JIMÉNEZ DÍAZ	Madrid
CLÍNICA MILLENIUM-DENT	Barcelona
HOSPITAL GENERAL UNIVERSITARIO GREGORIO MARAÑÓN	Madrid

Table S2. Biopsy evolution among the visits

	Visit 2 vs Baseline (n=26)	Visit 3 vs Baseline (n=13)	Visit 3 vs Visit 2 (n=11)
**Improvement**	4 (15.4)	3 (23.1)	2 (18.2)
**No change**	20 (77.4)	9 (69.2)	7 (63.6)
**Worsening**	2 (7.2)	1 (7.7)	2 (18.2)

Percentages values (in brackets) were calculated from patients in which a biopsy was performed in both compared visits.

Visit 2: 6 months; Visit 3: 12 months.
